# Preoperative botulinum A toxin as an adjunct for abdominal wall reconstruction: a single-center early experience at an Academic Center in New York

**DOI:** 10.1590/0100-6991e-20213152

**Published:** 2022-02-18

**Authors:** COSMAN CAMILO MANDUJANO, DIEGO LAURENTINO LIMA, ANALENA ALCABES, PATRICIA FRIEDMANN, XAVIER PEREIRA, FLAVIO MALCHER

**Affiliations:** 1 - Montefiore Medical Center, Department of Surgery - The Bronx - New York - United States

**Keywords:** Hernia, Botulinum A Toxin, Robotic Surgery, Laparoscopic Surgery, Hérnia, Toxina Botulínica A, Cirurgia Robótica, Cirurgia Laparoscópica

## Abstract

**Introduction:**

the botulinum toxin A (BTA) has been used to achieve a chemical component separation, and it has been used with favorable outcomes for the repair of complex ventral hernia (CVH) with and without loss of domain (LD). The aim of this study is to describe our early experience with the chemical component separation in the United Sates*.*

**Methods:**

a retrospective observational study of all patients who underwent ventral hernia repair for CVH with or without LD between July 2018 and June 2020. Preoperative BTA was injected in all patients via sonographic guidance bilaterally, between the lateral muscles to achieve chemical denervation before the operation. Patient demographics, anatomical location of the hernia, perioperative data and postoperative data are described.

**Results:**

36 patients underwent this technique before their hernia repair between July 2018 to June 2020. Median age was 62 years (range 30-87). Median preoperative defect size was 12cm (range 6-25) and median intraoperative defect size was 13cm (range 5-27). Median preoperative hernia sac volume (HSV) was 1338cc (128-14040), median preoperative abdominal cavity volume (ACV) was 8784cc (5197-18289) and median volume ration (HSV/ACV) was 14%. The median OR time for BTA administration was 45 minutes (range 28-495). Seroma was the most common postoperative complication in 8 of the patients (22%). Median follow up was 43 days (range 0-580).

**Conclusion:**

preoperative chemical component separation with BTA is a safe and effective adjunct to hernia repair in CVH repairs where a challenging midline fascial approximation is anticipated.

## RESUMO

**Introdução:**

a toxina botulínica A (TBA) tem sido usada para alcançar a separação química dos componentes, com resultados favoráveis para o reparo de hérnias ventrais complexas (HVC) com ou sem perda de domínio (PD). O objetivo deste estudo é descrever nossa experiência inicial com a separação química dos componentes nos Estados Unidos.

**Métodos:**

estudo retrospectivo observacional com todos os pacientes submetidos a reparo de hérnia ventral complexa com ou sem PD entre julho de 2018 e junho de 2020. A TBA pré-operatória foi injetada em todos os pacientes, guiada por ultrassonografia, bilateralmente entre os músculos laterais para alcançar a denervação química antes da operação. Dados demográficos dos pacientes, local da hérnia, dados peri-operatórios e pós-operatórios são descritos.

**Resultados:**

36 pacientes foram submetidos a esta técnica antes do reparo da hérnia. A idade mediana foi 62 anos (30-87). O tamanho mediano pré-operatório do defeito herniário foi 12cm (6-25) e o defeito mediano intra-operatorio foi 13cm (5-27). O volume mediano do saco herniário pré-operatório foi 1.338mL (128-14.040), o volume mediano da cavidade abdominal pré-operatório foi 8.784 (5.197-18.289) mL e a razão dos volumes foi 14%. A duração mediana da operação para aplicação da TBA foi 45 minutos (28-495). O seroma foi a complicação mais comum no pós-operatório, em oito pacientes (22%). O seguimento pós-operatório mediano foi de 43 dias (0-580).

**Conclusão:**

a separação pré-operatória de componentes com TBA é tratamento adjuvante seguro e eficaz em reparos de HVC em que aproximação da fáscia é antecipadamente desafiadora.

## INTRODUCTION

The repair of complex ventral hernias (CVH) poses a challenge to the general surgeon. Large hernia defects, loss of domain (LD), multiple previous operative repairs, and associated patient comorbidities can result in increased morbidity and high recurrence rates. The advances in surgical techniques and prosthetic materials, as well as improved patient selection and preoperative optimization, have led to a decrease in the morbidity of this patient population^1^.

One new adjunct option to help deal with this challenging patient population has been the use of botulin toxin type A (BTA). This neurotoxic agent blocks the release of acetylcholine from peripheral cholinergic nerve terminals inducing muscle paralysis^2^ ^,^ ^3^. Preoperative chemo-denervation of the abdominal wall muscles with BTA allows for fascial approximation, often reducing the need for additional surgical myofascial release due to the thinning and lengthening of the abdominal wall musculature achieved with BTA. The increased compliance of the abdominal wall achieved with the use of BTA may reduce the potential risks of abdominal compartment syndrome, ventilatory restriction, and prolonged ileus among other described complications related to increased intrabdominal pressures^4^ ^-^ ^6^.

The use of BTA to achieve a chemical component separation has been previously reported outside the United States (Europe, Mexico, Australia) with favorable outcomes. In this manuscript, we sought to report our institution’s initial experience with the use of BTA in the management of complex abdominal wall reconstruction (AWR)^5^ ^,^ ^7^ ^-^ ^9^.

## METHODS

### Study Design

A descriptive, retrospective analysis of all patients undergoing BTA injection as an adjunct treatment for repair of a complex ventral hernia from July 2018 to June 2020 was conducted. Inclusion criteria was all adult patients with CVH or inguinoscrotal hernia who underwent BTA injection prior to hernia repair. CVH was defined by one or more of the following criteria stratified by defect size (>8cm in width) with or without LD plus one or more of the following criteria: laterality (lumbar, lateral and subcostal locations), presence of a parastomal hernia, wound classes III (contaminated) and IV (dirty/infected) (CDC wound classification system) and the condition of the soft tissues (full-thickness abdominal wall defects, loss of substance, denervated muscles, skin grafts, ulcers, open abdomen and other hernia conditions including significantly affected soft tissue)^10^ ^,^ ^11^. LD was defined as a ventral hernia large enough such that simple reduction in its contents and primary fascial closure either cannot be achieved without additional reconstructive techniques or cannot be achieved without significant risk of complications due to elevated intra-abdominal pressure^12^. For those patients with suspected LD, the Tanaka method was used to estimate the volume of the abdominal cavity and hernia sac and their ratios^13^. Patients with complex hernias were selected for preoperative chemo-denervation with BTA based on an abdominal wall specialist’s expertise. This determination was based on an expert’s judgment in anticipating difficult primary closure of a complex hernia defect.

Data were retrieved from electronic medical records (EMR) from an academic medical center. This study was approved by the Institution Review Board of the institution and all HIPAA compliant mechanisms were followed.

### Data Collection

Data was retrospectively collected and divided into the following sections: patient characteristics, hernia characteristics, BTA data, perioperative data, and patient outcomes. 

Preoperative distances between rectus abdominis muscles as well as hernia sac/peritoneal volumes for patients with LD were measured based on preoperative computerized tomography (CT) scan and were reported when available. 

### BTA protocol

**Figure 1:**
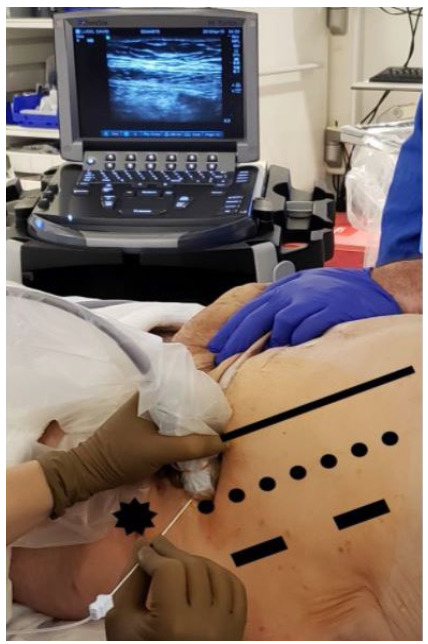
Ultrasound-guided BTA injection demonstrating a view of the left lateral abdominal wall demonstrating the landmarks for the injection sites. Anterior Superior Iliac Crest (Black Star), Semilunaris line (Black Line), Anterior Axillary line (dotted line) Mid Axillary line (Black Rectangles).

In our practice, we utilize 200 Units of Botox® (diluting 200U in 30mL of normal saline)^5^. We inject under ultrasonography (US) guidance in the plane between the internal oblique and transversus abdominis muscles. Five mL of BTA are injected over three points on each side of the abdominal wall between the anterior axillary line and the midclavicular line, and between the costal margin, and the superior iliac crest as listed in Table 2 and depicted in Figure 1. Every injection site is confirmed with saline injection “bubble sign” on US. Injections were all performed by the same surgeon in an outpatient surgery setting under conscious sedation. The patients were subsequently discharged home and returned electively for their planned AWR within 4-6 weeks.

### Preoperative Progressive Pneumoperitoneum (PPP) Protocol

Selection parameters for patients who underwent PPP was determined by the presence of LD as defined above by Tanaka Scores >25%. A diagnostic laparoscopy was performed with optical trocar at left Palmer’s point. After the creation of the pneumoperitoneum, a central line catheter was inserted in the right upper quadrant under direct vision by the Seldinger technique. The catheter was positioned on the top of the liver, avoiding the hernia sac. Once in the cavity, the catheter was sutured to the skin and a three-way key and antibacterial filter is placed in the distal lumen. We introduced usually over 1000mL of ambient air per day throughout 10 days and monitored the patient for symptoms such as nausea, dyspnea and abdominal pain. Patients were at the hospital during the whole procedure and had prophylactic anti-coagulation. After completing the insufflation, the new volumes and diameters were evaluated again by a CT scan. We included patients also undergoing PPP to demonstrate the versatility of the use of BTA in this patient population.

### Statistical analysis

Descriptive analysis was performed. Categorical variables were expressed as counts and percentiles. Continuous variables with skewed distributions were reported as median and range. Data were analyzed with SAS v9.4.

## RESULTS

A total of 36 patients underwent the BTA technique before their hernia repair between July 2018 to June 2020. Median age was 62 years (range 30-87) and median BMI 33kg/m^2^ (range 17-48kg/m^2^). Seven patients (19%) were active smokers and 9 had diabetes mellitus (25%). Patients’ demographics are listed in Table 1. 

**Table 1:** Patient demographics.

Total=36	n (%)
Sex	
Male	20 (56)
Female	16 (44)
Median age (range)*	62 (30-87)
Median BMI (range)**	33 (17-48)
Race	
Hispanic	16 (44)
White	8 (22)
Black	4 (11)
Asian	1 (3)
Other	7 (19)
ASA	
Class II	9 (25)
Class III	26 (72)
Class IV	1 (3)
Comorbidities	
Diabetes Mellitus	9 (25)
Smoking	7 (19)
COPD	5 (14)
CHF	3 (8)
History of previous abdominal surgery	
Laparotomy	26 (72)
Laparoscopy	2 (6)
Both	8 (22)

*in years

**kg/m ^*2*^

*BMI: body mass index; COPD: chronic obstructive pulmonary disease; CHF: chronic heart failure; ASA: american society of anesthesiologists.*

Thirty-five patients had complex ventral hernias and one patient had a giant inguinoscrotal hernia. LD was present in 29 patients (81%). Median preoperative defect size was 12cm (range 6-25) and median intraoperative defect size was 13cm (range 5-27). Median preoperative hernia sac volume (HSV) was 1,338mL (128-14,040), median preoperative abdominal cavity volume (ACV) was 8,784mL (5,197-18,289) and median volume ration (HSV/ACV) was 14% (Table 2).

**Table 2:** Hernia Characteristics.

Total=36	n (%)
Hernia type	
Primary Ventral Hernia	27 (75)
Incisional Hernia	6 (17)
Incisional + Parastomal Hernia	2 (6)
Inguinal Hernia	1 (3)
Loss of Domain	29 (81)
Median preoperative defect size (range)*	12 (6-25)
Median Intraoperative defect size (range)*	13 (5-27)
Median preoperative distance between Rectus muscles (range)*	11 (0-36)
PPP	6 (17)
Hernia volumes and ratios	
Median Preoperative Hernia Sac Volume (HSV) (Range)**	1,338 (128-14,040)
Median Preoperative Abdominal Cavity Volume (ACV) (Range)**	8,784 (5,197-18,289)
Median Preoperative Volume Ratio (VR) (Range)	14% (1-76)
Laparoscopia	2 (6)
Both	8 (22)

*Width in cm

**in cc

*PPP: preoperative progressive pneumoperitoneum.*

All patients underwent preoperative US-guided BTA injection in the outpatient setting, receiving a total of 200U (100U per side). BTA administration data are as listed in Table 3. The median time from BTA administration to AWR was 33 days (range 0-103d). The median OR time for BTA administration was 45 minutes (range 28-495 minutes). The median distance between the rectus abdominis muscles measured preoperatively on cross-sectional imaging was 11cm (range 0-36cm). Six patients (17%) underwent PPP before AWR. Fascial closure was achieved in all cases and component separation technique was used in 17 patients (47%) (Table 4). 

**Table 3:** BTA administration.

Total units administered per patient	200U
Units administered per laterality	100U
Dilution of BTA	200U in 30ml of normal Saline
Anatomical points of BTA administration	
Mid Axillary Line between Rib Margin and Superior Iliac Crest	3 points
Volume administered per point	5ml
Median time from Botox administration to AWR (days) (Range)	33 (0-103)
Median OR time of BTA administration (minutes) (Range)	45 (28-495)

BTA: botulinum toxin A; OR: operative room; AWR: abdominal wall reconstruction.

**Table 4:** Surgical outcomes.

Total=36	n (%)
Median Surgical time (range)*	296 (139-567)
Median EBL (range)**	100 (20-500)
Fascial closure (ventral)	35 (100)***
Operative technique	
Primary tissue repair	10 (28)
Primary tissue repair + Mesh	28 (78)
CST	17 (47)
Mesh characteristics	
Polypropylene	23 (64)
Phasix	7 (19)
Bio-A	1 (3)
Synecor	1 (3)
Median mesh area in cm^2^ (range)	775 (36-1,330)
Mesh position	
Onlay	18 (50)
Sublay	13 (36)
Intra-peritoneal	2 (5.6)
Wound classification	
1	22 (61)
2	7 (19)
3	6 (17)
4	1 (3)
Associated procedures	
Panniculectomy	14 (42)
Intestinal Resection	5 (14)
Cholecystectomy	4 (12)
Enterocutaneous Fistula Takedown	3 (9)
Ostomy Reversal	3 (9)
Appendectomy	2 (6)
Hysterectomy	1 (3)
Sleeve Gastrectomy	1 (3)
Gastric Bypass	1 (3)
Scrotoplasty/Orchiectomy	1 (3)
Splenectomy	1 (3)

*in minutes

**in ml

***excluded the inguinal hernia repair

*EBL: estimated blood loss; CST: component separation technique; IPOM: intraperitoneal onlay mesh.*

Mesh reinforcement was used in 28 patients (78%). Twenty-three patients (64%) had a polypropylene mesh, 7 patients (19%) with a P4HB mesh, one patient had a biological mesh and another a Synecor mesh. In a few cases, a P4HB coated mesh was used as a protection so the polypropylene mesh would not be in contact with the viscera. Operative data are as listed in Table 4. Seroma was the most common postoperative complication in 8 of the patients (22%). Median LOS was 5 days (range 0-66) and median follow up was 43 days (range 0-580). (Table 5).

**Table 5:** Postoperative outcomes.

Total=36	n (%)
Seroma	8 (22)
Ileus	6 (17)
Post-Operative Intubation	5 (14)
SSI	5 (14)
Acute Renal Failure	3 (8)
Enterotomy	3 (8)
Death	2 (6)
Wound Dehiscence	2 (6)
Enterocutaneous Fistula	1 (3)
Bladder Injury	1 (3)
Median ICU stay (days) (Range)	0 (0-25)
Median time to Extubation (Days) (Range)	0 (0-3)
Median Length of Stay (Days) (Range)	5 (0-66)
Median Follow Up Time (Days) (Range)	43 (0-580)
Recurrence	0

*SSI: surgical site infection; ICU: intensive care unit.*

## DISCUSSION

Complex ventral hernia repair is challenging for surgeons. BTA is one of several developed techniques which allows the surgeon to achieve primary fascial closure. In our study, we achieved fascial closure in all our patients with CVH. Six patients also had PPP prior to surgery. Our results are similar to what has been published in the literature. 

**Figure 2A:**
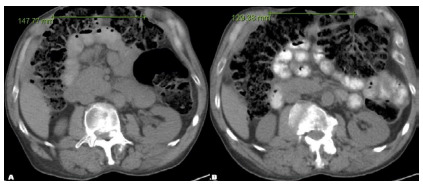
Demonstrates a large anterior abdominal wall defect measuring 14.7cm in its transverse diameter before BTA Administration. Figure 2B. Demonstrates the anatomical changes in the abdominal wall configuration four weeks after administration of BTA with a mean reduction of the transverse diameter of the ventral defect by roughly 20,cm.

Ibarra-Hurtado et al. first reported use of BTA for abdominal wall hernias in 2009. Their initial experience included 12 patients with complex ventral hernias after trauma laparotomies, reporting a decrease in mean transverse defect size four weeks after BTA administration of 5.25cm with an 100% fascial closure rate. They expanded on this initial experience in 2014 with an additional 17 patients with similarly complex incisional hernias^7^ ^,^ ^14^. The effect of BTA was associated with both a thinning of the abdominal wall musculature by an average of 1.0cm and lengthening of the abdominal wall musculature by an average of 2.52cm per side. We found similar effects in the abdominal wall configuration of our patient population, as depicted in Figure 2. More recently, in 2015 and 2017, Bueno-LLedó et al. reported their BTA experience plus the use of PPP for AWR in the setting of large incisional hernias (LIH) and LD^8^. 

**Figure 3A:**
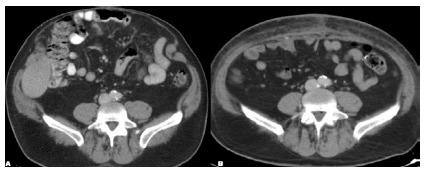
Preoperative image before administration of BTA evidencing a large wide-necked ventral hernia containing both small and large intestinal contents within the hernia. Figure 3B. Postoperative image following BTA administration, ventral hernia repair with bilateral component separation, and onlay polypropylene mesh reinforcement.

**Figure 4:**
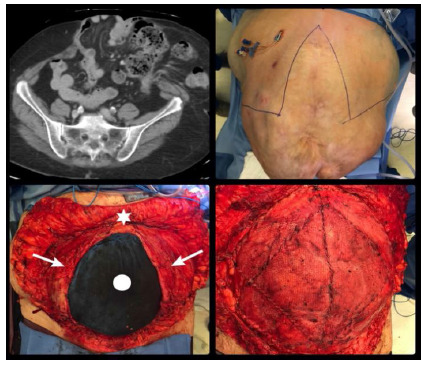
CT scan (top left) demonstrating a multicompartment large ventral hernia with loss of domain. Fleur de Lis incision (Top Right), Note the Right upper quadrant catheter utilized for preoperative PPP after BTA Injection. Completed dissection of the hernia sac (bottom left) with exposure of a 23x20cm defect (fascial edges marked by white arrows). The patient’s head is oriented superiorly (white star) while the abdominal contents are covered with a blue towel (white circle) - polypropylene onlay mesh reinforcement (bottom right) after primary fascial closure. Myofascial release was not necessary due to the effect of BTA and PPP.

*CT: computed tomography; PPP: preoperative progressive pneumoperitoneum; BTA: botulinum toxin A.

Chemo-denervation with BTA is an off-label use of the product prior to abdominal wall reconstruction to achieve temporary muscle paralysis. It allows for the reduction of the size of the hernia defect as well as thinning and lengthening of the abdominal wall musculature with partial reduction of the hernia sac contents into the abdominal cavity^14^. These effects facilitate fascial closure and in some cases, can obviate the need for additional myofascial release^15^. Thus, maintaining virgin planes for future repair in case of a recurrence. 

Different BTA injection protocols have been reported with doses varying from 500U with five injections per laterality, 300U with three injections per laterality or 200U with three injections per laterality^8^ ^,^ ^16^. A study by Field M et al. demonstrated that there are differences in the quantity of Neurotoxin (150kDa BTA) in each potency unit for each commercial product, with the highest amount of Neurotoxin and longest duration of response noted with Dysport^®3^.

It is important to note that the optimal time for intervention is not precisely known, but clinically, the effects become apparent in 2-3 days, with full effect by two weeks and decline after 11-12 weeks^15^. The lowest rate of primary fascial closure in the literature was reported by Zendajas et al., which is likely explained by the early intervention after administration of BTA in their protocol (they injected BTA on the day of the operation for most of their patients, and averaged six days from injection to surgical procedure in their remaining patients)^6^. In our study, the average time from BTA injection to the operation was 37.5 days with our earliest intervention at six days post-injection of BTA with no clinically noticeable difference in the outcome, however we did not run statistical comparisons. In our practice, we typically allow a time interval of four weeks before undergoing the operation. We report our mean operative time for BTA administration and the time interval between BTA administration and AWR to demonstrate the feasibility of our approach. Currently, BTA time interval and ideal dose remain a subject of debate despite reported outcomes in the literature. 

Our mean transverse defect size intraoperatively measured following the administration of BTA did not have a reduction from preoperative CT measurements. We have to take into consideration that our intraoperative measurements were performed following our initial dissection, which is biased towards having more substantial defects after completion of our dissection. Primary fascial closure was achieved in all cases. The need for additional myofascial release was determined intraoperatively and was not necessary in half of our patients. In comparison to our experience, Ibarra Hurtado et al reported the need for myofascial release in six patients (50%) out of 12 patients undergoing BTA injection for AWR in a case series in 2012. They followed their initial experience with a case series of 17 trauma patients that same year, who had undergone AWR at least 12 months after management of an open abdomen requiring myofascial release in 13 patients (76%)^7^ ^,^ ^14^. Rodriguez Acevedo et al. published a retrospective case series of 56 patients undergoing laparoscopic AWR after BTA injection performing myofascial release in 11 patients (19%)^5^. Bueno LLedó et al. performed myofascial release in all their patients in a retrospective series of 70 patients with LD undergoing open AWR after BTA injection and PPP^8^.

Our operative time was influenced by the concomitant procedures required in the interdisciplinary management of these complex hernias. We report two deaths in our sample. One was a 38 years-old male who underwent extensive small bowel resection alongside hernia repair and died due to complications from sepsis (fungemia not related to the abdominal operation). The other death was a 77 years-old male who died due to complications after a PEG tube placement.

### Limitations of the Study

Our study is limited by our small sample size, and by the fact that it is only a case series with a single institution experience and no control group. We did not calculate sample size as this is an innovative technique and we aimed to show our early experience with all patients who underwent BTA injection prior to hernia repair. Our results may not be representative of other patient populations or surgeon expertise. Also, we have a short follow-up which does not allow us to properly evaluate recurrence. Despite this, our study is an addition to the literature on the use of BTA, which while limited, is growing with reports of favorable outcomes and safety profiles, which has driven innovative approaches that could be measured at a larger scale in a prospective randomized controlled fashion.

## CONCLUSION

AWR in the setting of CVH with or without LD, is a challenging problem. Preoperative chemo-denervation with BTA may be a valuable adjunct for complex AWR, often limiting the need for additional myofascial release and mitigating the effect and complications associated with increased intrabdominal pressures. The safety and efficacy of these surgical adjuncts need to be validated with larger prospective studies.
